# Supplementary material for: Sex-specific developmental gene expression atlas unveils dimorphic gene networks in C. elegans
Source: Nat Commun. 2024 May 20;15:4273. doi: 10.1038/s41467-024-48369-z (PMC11106331; doi:10.1038/s41467-024-48369-z)
Supplement: Supplementary file 1 — Supplementary Information [file 41467_2024_48369_MOESM1_ESM.pdf]

SUPPLEMENTARY INFORMATION

Sex-Specific Developmental Gene Expression Atlas Unveils Dimorphic Gene Networks in *C. elegans*

This file includes nine supplementary figures.

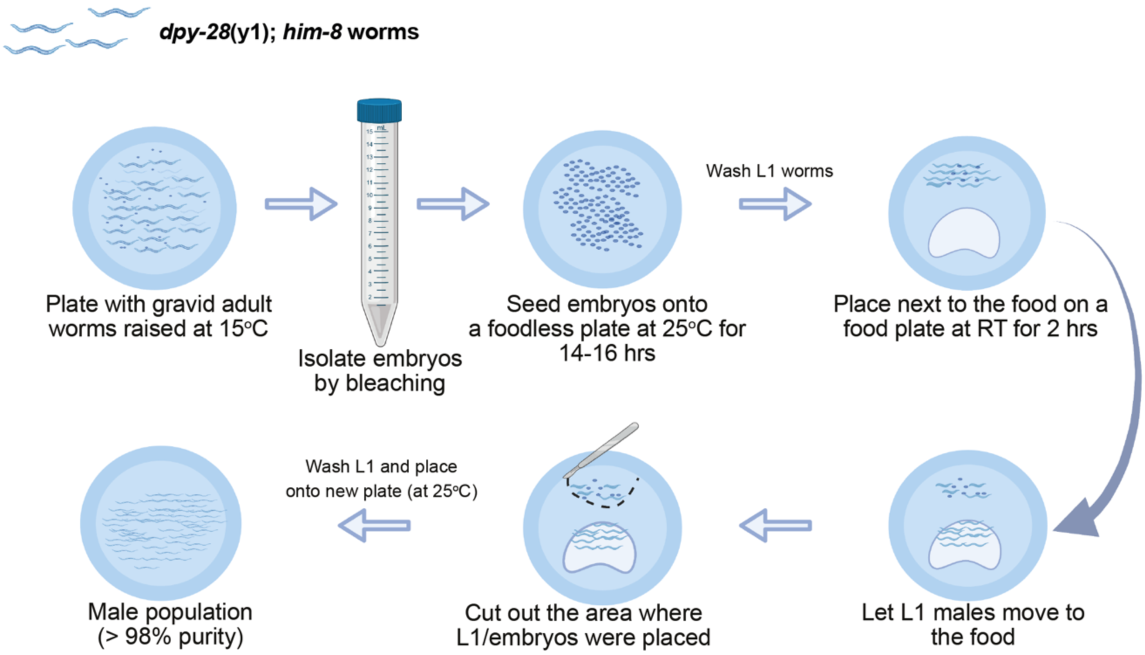

Supplementary Fig. 1. Protocol for large-scale male isolation in the early larval stage of *C. elegans*. Created with Biorender.com.

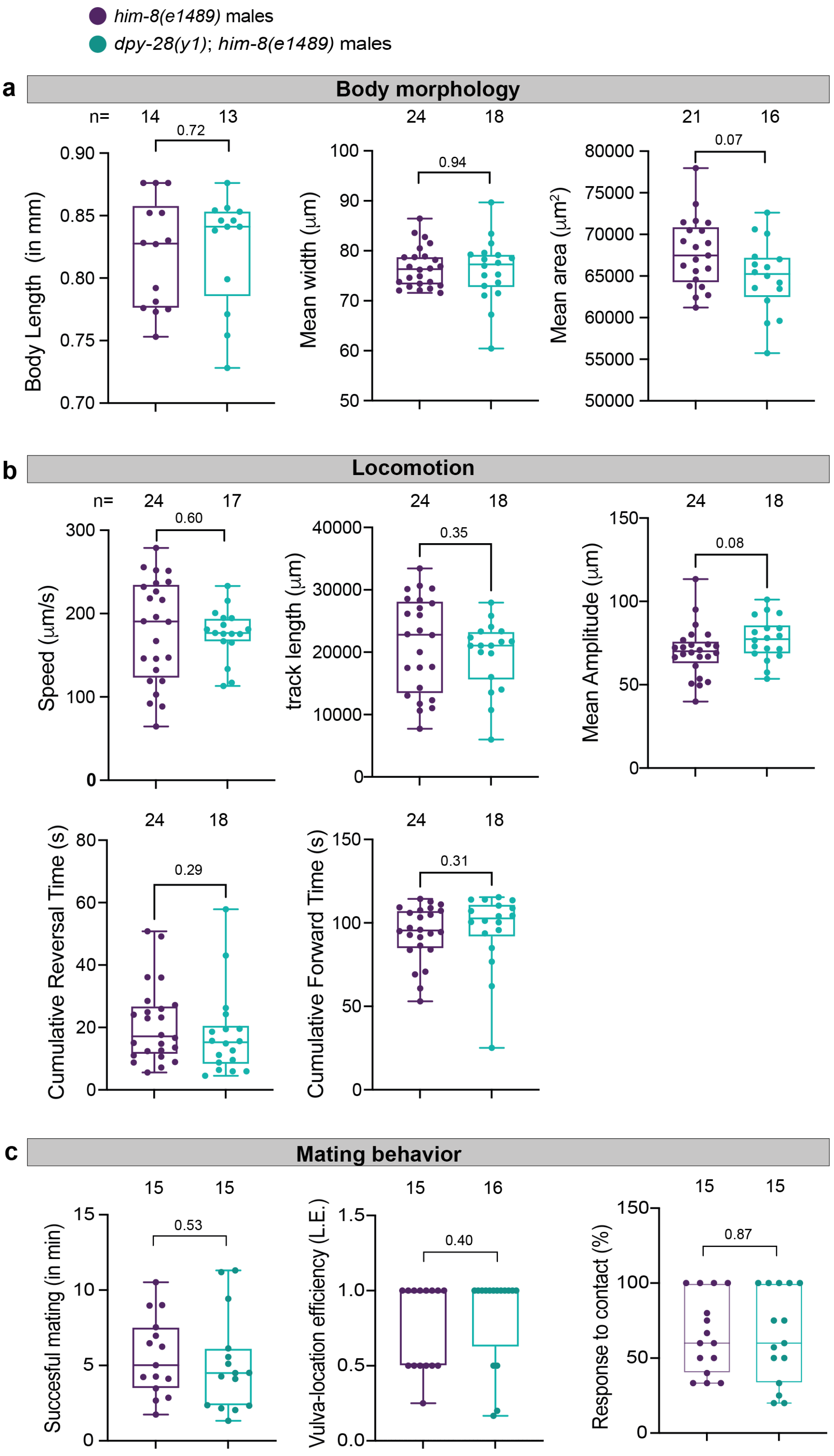

him-8(e1489) males
dpy-28(y1); him-8(e1489) males
a
Body morphology
n=
14
13
0.72
Body Length (in mm)
24
18
0.94
Mean width (µm)
21
16
0.07
Mean area (µm²)
b
Locomotion
n=
24
17
0.60
Speed (µm/s)
24
18
0.35
track length (µm)
24
18
0.08
Mean Amplitude (µm)
24
18
0.29
Cumulative Reversal Time (s)
24
18
0.31
Cumulative Forward Time (s)
c
Mating behavior
15
15
0.53
Succesful mating (in min)
15
16
0.40
Vulva-location efficiency (L.E.)
15
15
0.87
Response to contact (%)

Supplementary Fig. 2. *dpy-28* males are identical to wild-type males. a Quantification of body morphology of *dpy-28*(*y1*);*him-8*(*e1489*) males and *him-8*(*e1489*) males. **b** Quantification of locomotion. **c** Quantification of mating behavior. In the box-and-whiskers graph, the center line in the box denotes the median, while the box contains the 25th to 75th percentiles of the dataset, whiskers define the minimum and maximum value with dots showing all points. We performed a two-sided Mann-Whitney test for all comparisons, ns- non-significant. The number of animals is indicated above each bar graph.

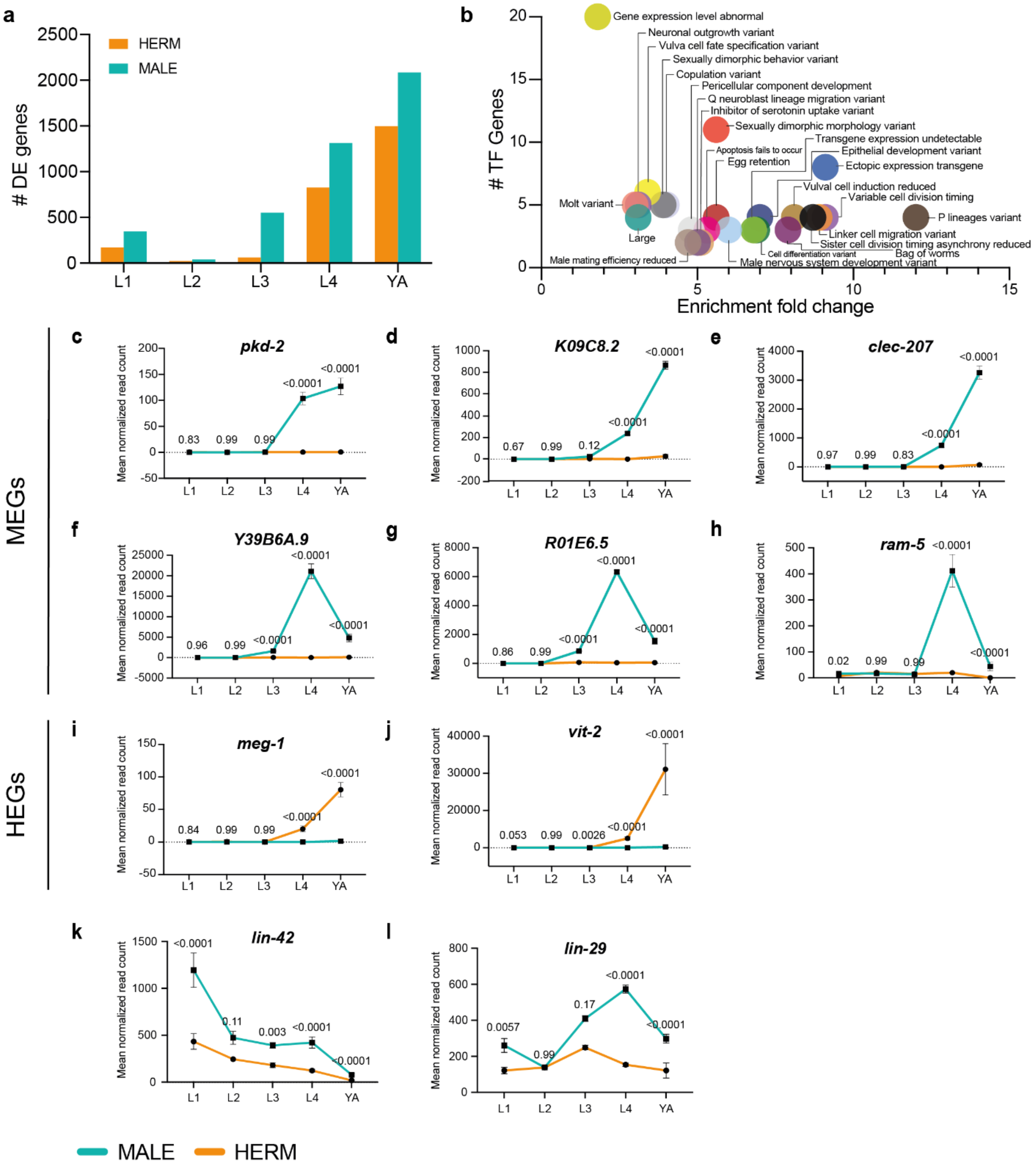

MEGs- Male enriched genes
HEGs- Hermaphrodite enriched genes

Supplementary Fig. 3. Expression of known sex-specific genes rises during or after sexual maturation. **a** Bar graph representing the number of differentially expressed genes across all the developmental stages in both sexes (cyan: males and orange: hermaphrodites). DEGs, differentially expressed genes**. b** Phenotype GO term enrichment analysis of differentially expressed transcriptional factors before sexual maturation (until L3). Each circle represents a GO term. The significance of the enrichment was analyzed using the q-value threshold of 0.1. TFs, transcription factors. **c-l** Normalized read counts of *pkd-2* (**c**), *K09C8.2* (**d**), *clec-207* (**e**), *Y39B6A.9* (**f**), *R01E6.5* (**g**), *ram-5* (**h**) *meg-1* (**i**) *vit-2* (**j**) *lin-42* (**k**) *lin-29* (**l**) across all the developmental stages in both the sexes (cyan: males and orange: hermaphrodites). **k-l** are two examples of TFs with a sexually dimorphic expression pattern early during development. n=4 biological repeats per sample for (c-l). Error bars are standard error of the mean (SEM). adjusted p-values were calculated by a two-sided Wald test for each comparison performed by DESeq2[1]**.**

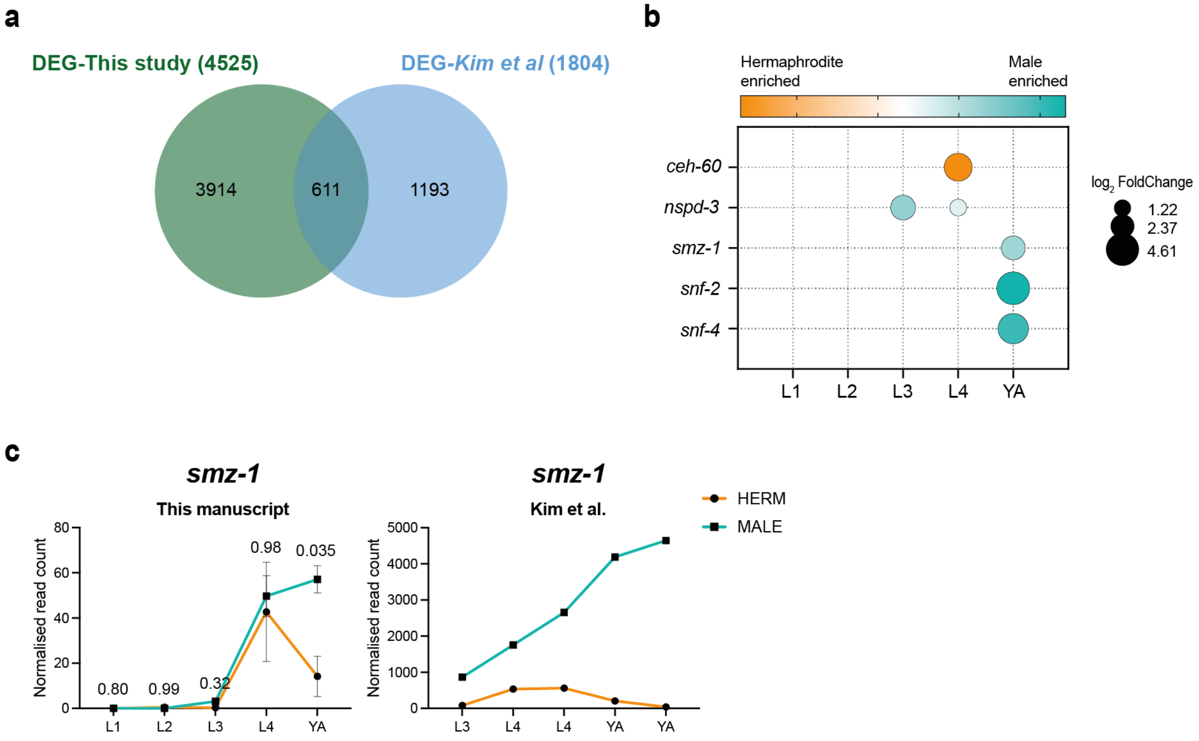

Supplementary Fig. 4. Data set comparison. **a** Venn diagram comparing differentially expressed genes in both sexes from this study with *kim et al*, 2016[2]. DEGs, differentially expressed genes**. b** Bubble plot representation of *ceh-60*, *nspd-3*, *smz-1*, *snf-2,* and *snf-4* genes differentially expressed in any of the five developmental stages of the two sexes. Bubble size represents log2 of fold change in expression of that gene (only genes that passed the filter padj <= 0.05, |log2 fold change| >= 1 and basemean >= 5 are plotted), and bubble color represents enrichment in either sex (male enrichment in cyan, hermaphrodite enrichment in orange). Error bars are standard error of the mean (SEM). **c** Normalized expression values of *smz-1* across the experimental stages (late L3 to YA) in both sexes from this manuscript, n=4 biological repeats per sample and *kim et al.*, 2016 (cyan: males, orange: hermaphrodites). Adjusted p-values were calculated by a two-sided Wald test for each comparison performed by DESeq2[1].

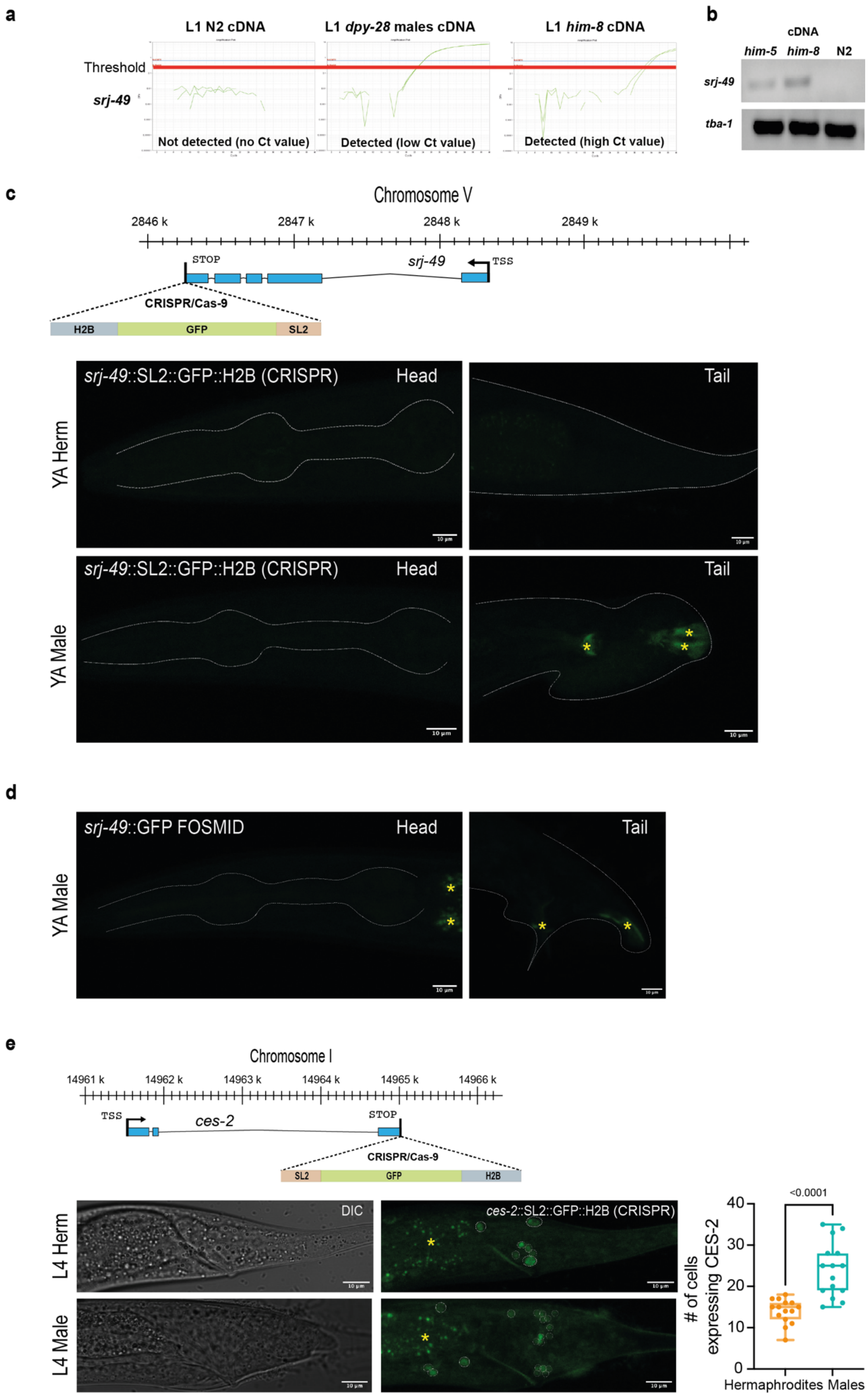
a
L1 N2 cDNA
L1 dpy-28 males cDNA
L1 him-8 cDNA
Threshold
srj-49
Not detected (no Ct value)
Detected (low Ct value)
Detected (high Ct value)
b
cDNA
him-5
him-8
N2
srj-49
tba-1
c
Chromosome V
2846 k
2847 k
2848 k
2849 k
STOP
srj-49
TSS
CRISPR/Cas-9
H2B
GFP
SL2
srj-49::SL2::GFP::H2B (CRISPR)
Head
Tail
YA Herm
YA Male
d
srj-49::GFP FOSMID
Head
Tail
YA Male
e
Chromosome I
14961 k
14962 k
14963 k
14964 k
14965 k
14966 k
TSS
ces-2
STOP
CRISPR/Cas-9
SL2
GFP
H2B
DIC
ces-2::SL2::GFP::H2B (CRISPR)
L4 Herm
L4 Male
<0.0001
# of cells expressing CES-2
0
10
20
30
40
Hermaphrodites
Males

Supplementary Fig. 5. ***srj-49*** **mRNA is detectable by RT-PCR only in strains containing high male ratios. a** Real-time qPCR amplification curve of *srj-49* mRNA at L1 stage of male-enriched strains (*him-8* and *dpy-28*(*y1*);*him-8* males) and wild-type N2 hermaphrodites-containing control strain of *C. elegans*. Each line (green) represents a biological repeat. **b** DNA electrophoresis of PCR products amplified using *srj-49* and *tba-1* (tubulin) primers from cDNA isolated from mixed populations of *him-5*, *him-8,* and N2 plates. *srj-49* mRNA is only detected in strains containing male populations and not detected in wildtype N2, which typically contains only hermaphrodites. **c** Top, schematic of the CRISPR/Cas9 genome editing strategy used to engineer the *srj-49(syb4956)* CRISPR reporter. Bottom, representative confocal micrographs of head and tail of young adult hermaphrodite and head and tail male carrying the SRJ-49::GFP fosmid. The head pharynx and tail are outlined in a white dashed line. Autofluorescence is indicated by a yellow asterisk mark. Scale bars represent 10µm. **d** Representative confocal micrographs of the head and tail of a young adult male right carrying the *srj-49(syb4956)* reporter expression. The head pharynx and tail are outlined in a white dashed line. Autofluorescence is indicated by a yellow asterisk mark. Scale bars represent 10µm. **e** Top, schematic of the CRISPR/Cas9 genome editing strategy used to engineer the *ces-2(syb4992)* reporter expression. Bottom left, representative confocal micrographs of the expression pattern of *ces-2(syb4992[ces-2::SL2::GFP::H2B])* at the L4 stage in the tail of both sexes. Bottom right, quantification of the number of *ces-2(syb4992)* GFP-expressing cells, Bottom left. n=15 worms per group. The head pharynx and tail are outlined in a white dashed line. Autofluorescence is indicated by a yellow asterisk mark. Scale bars represent 10µm. In the box-and-whiskers graph, the center line in the box denotes the median, while the box contains the 25th to 75th percentiles of the dataset, whiskers define the minimum and maximum value with dots showing all points. We performed a two-sided Mann-Whitney test for each comparison.

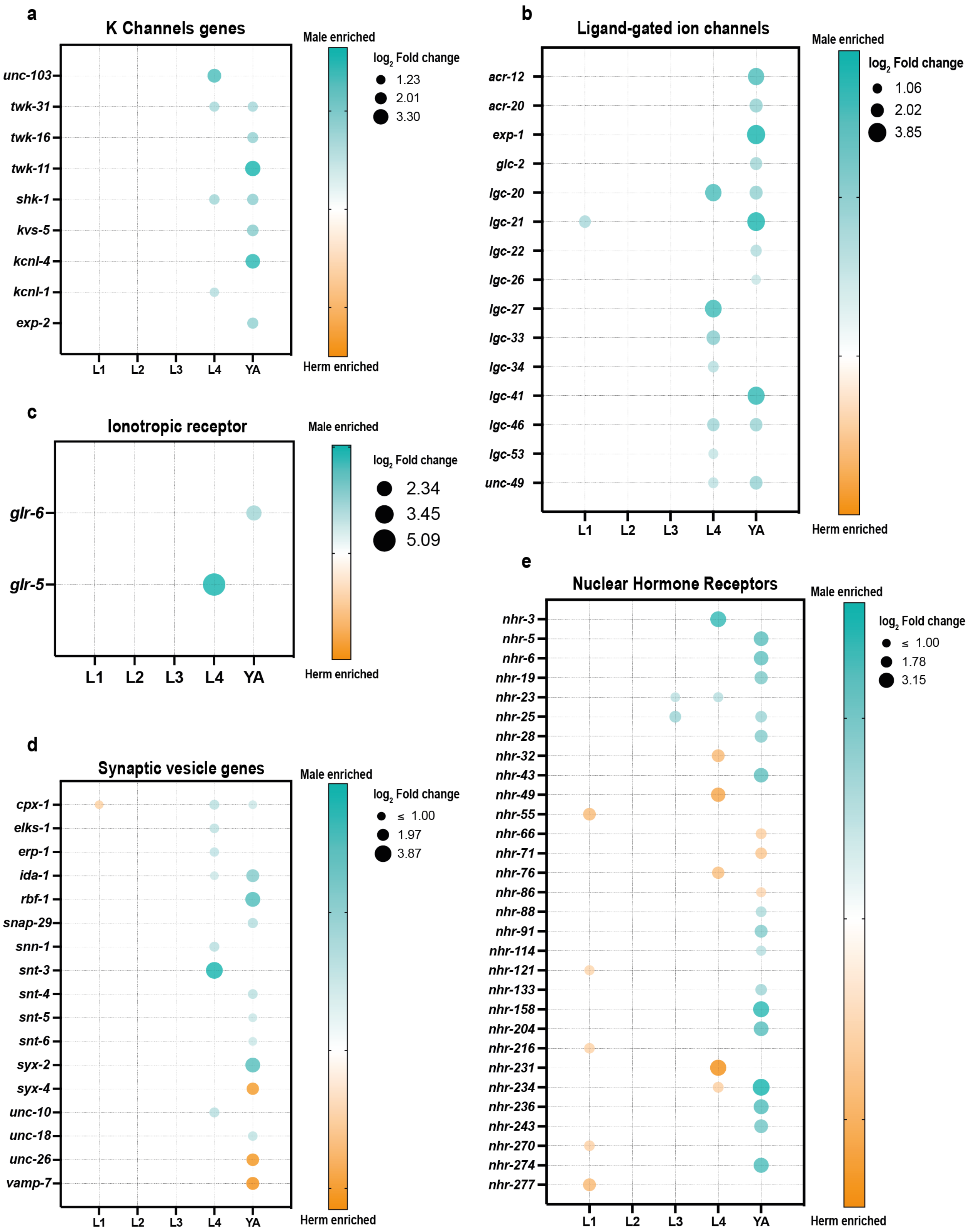

Supplementary Fig. 6. Neuronal gene families with sexually dimorphic regulation. **a-e** Bubble plot representation of K channel (**a**), Ligand-gated ion channels (**b**), Ionotropic receptors (**c**),

synaptic vesicle genes (**d**), and Nuclear hormone receptors (**e**) genes differentially expressed in any of the five developmental stages of the two sexes. Bubble size represents log2 of fold change in expression of that gene (only genes that passed the filter padj <= 0.05, |log2 fold change| >= 1 and basemean >= 5 are plotted), and bubble color represents enrichment in either sex (male enrichment in cyan, hermaphrodite enrichment in orange).

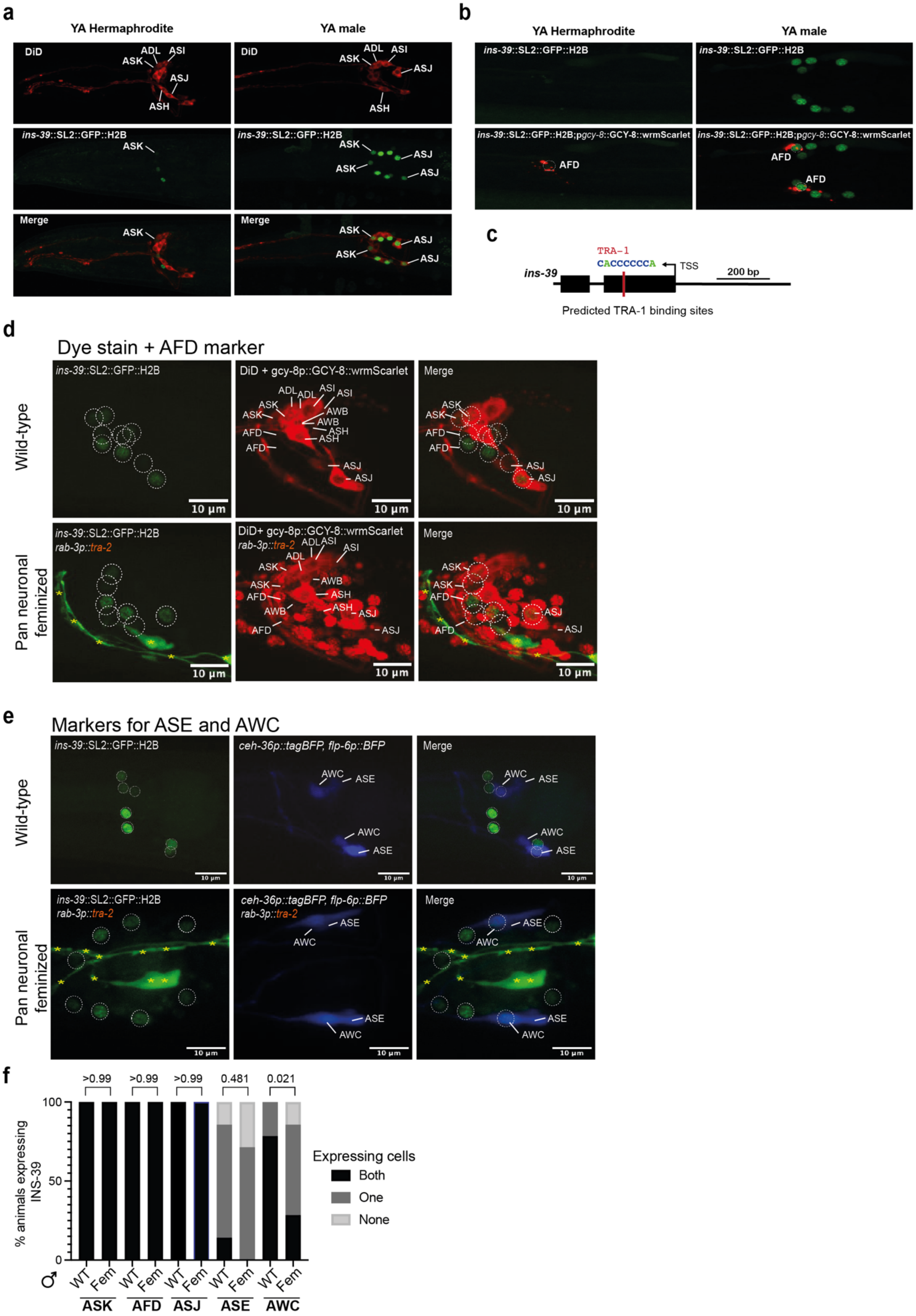

a
YA Hermaphrodite
YA male
DiD
ADL
ASI
ASK
ASJ
ASH
ins-39::SL2::GFP::H2B
Merge
b
pgcy-8::GCY-8::wrmScarlet
AFD
c
TRA-1
CACCCCCCA
TSS
ins-39
200 bp
Predicted TRA-1 binding sites
d
Dye stain + AFD marker
Wild-type
Pan neuronal feminized
DiD + gcy-8p::GCY-8::wrmScarlet
rab-3p::tra-2
AWB
10 µm
e
Markers for ASE and AWC
ceh-36p::tagBFP, flp-6p::BFP
AWC
ASE
f
% animals expressing INS-39
>0.99
0.481
0.021
100
50
0
WT
Fem
ASK
AFD
ASJ
ASE
AWC
Expressing cells
Both
One
None

Supplementary Fig. 7. Pan-neuronal feminization affects INS-39 expression specifically in AWC, and to a lesser extent in ASE neurons. a Representative confocal micrographs of a young adult hermaphrodite and male expressing the *ins-39(syb4915)* reporter and co-stained with Vybrant lipophilic dye (DiD), enabling identification of sensory amphid neurons. Scale bars represent 10μm. **b** Representative confocal micrographs of a young adult hermaphrodite and male expressing the *ins-39(syb4915)* reporter with AFD marker. Scale bar 10μm. **c** Schematic representation of predicted TRA-1 transcription factor binding site on the exonic region of *ins-39*. The binding site was determined using RSAT matrix-scan search tool (see methods). **d** Representative confocal micrographs of the *ins-39(syb4915)* reporter expression in a wild-type male and pan-neuronally feminized male, expressing *rab-3p::tra-2(IC)* and co-stained with Vybrant lipophilic dye (DiD), enabling identification of sensory amphid neurons. Co-injection marker is indicated by a yellow asterisk mark. Scale bars represent 10μm. **e** Representative confocal micrographs of the *ins-39(syb4915)* reporter expression in a wild-type male and pan-neuronally feminized male, expressing *rab-3p::tra-2(IC)* with ASE and AWC (*ceh-36p::tagBFP; flp-6p::BFP*) markers enabling identification of sensory amphid neurons. Co-injection marker is indicated by a yellow asterisk mark. Scale bars represent 10μm. **f** Quantification of the % of animals expressing INS-39 in the relevant sensory neurons. For calculating statistics, worms were grouped into full expression (both neurons) and partial expression (0 or 1 neuron), and statistical analysis was calculated using a two-sided Fisher's exact test. n=14 for both groups.

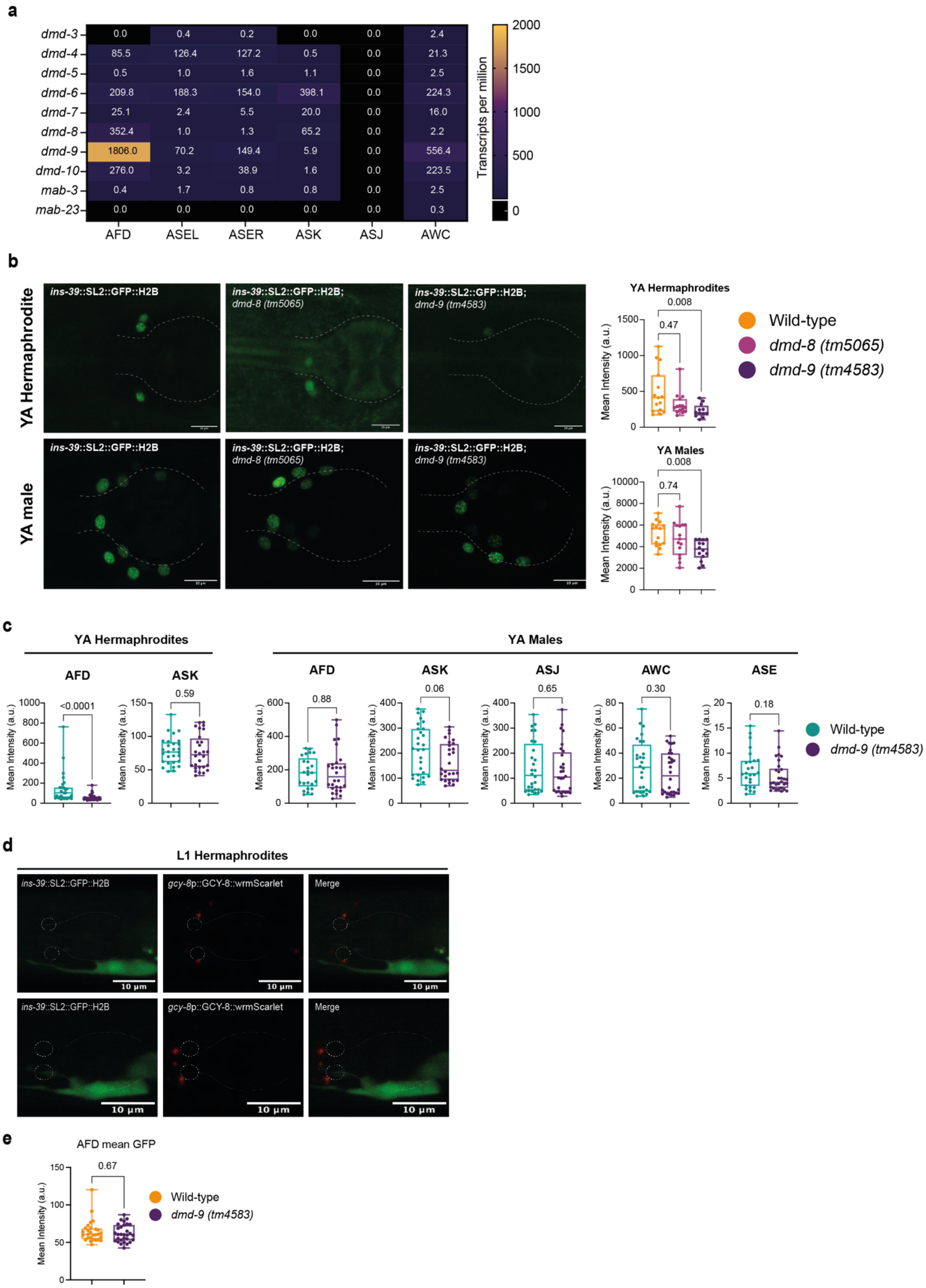
a
dmd-3 0.0 0.4 0.2 0.0 0.0 2.4
dmd-4 85.5 126.4 127.2 0.5 0.0 21.3
dmd-5 0.5 1.0 1.6 1.1 0.0 2.5
dmd-6 209.8 188.3 154.0 398.1 0.0 224.3
dmd-7 25.1 2.4 5.5 20.0 0.0 16.0
dmd-8 352.4 1.0 1.3 65.2 0.0 2.2
dmd-9 1806.0 70.2 149.4 5.9 0.0 556.4
dmd-10 276.0 3.2 38.9 1.6 0.0 223.5
mab-3 0.4 1.7 0.8 0.8 0.0 2.5
mab-23 0.0 0.0 0.0 0.0 0.0 0.3
AFD ASEL ASER ASK ASJ AWC
Transcripts per million
2000 1500 1000 500 0
b
YA Hermaphrodite
YA male
ins-39::SL2::GFP::H2B
ins-39::SL2::GFP::H2B; dmd-8 (tm5065)
ins-39::SL2::GFP::H2B; dmd-9 (tm4583)
YA Hermaphrodites
YA Males
Mean Intensity (a.u.)
0.008
0.47
0.74
Wild-type
dmd-8 (tm5065)
dmd-9 (tm4583)
c
YA Hermaphrodites
YA Males
AFD
ASK
ASJ
AWC
ASE
<0.0001
0.59
0.88
0.06
0.65
0.30
0.18
Mean Intensity (a.u.)
Wild-type
dmd-9 (tm4583)
d
L1 Hermaphrodites
ins-39::SL2::GFP::H2B
gcy-8p::GCY-8::wrmScarlet
Merge
10 µm
e
AFD mean GFP
0.67
Mean Intensity (a.u.)
Wild-type
dmd-9 (tm4583)

Supplementary Fig. 8. *dmd-9* regulates INS-39 expression in AFD neurons in hermaphrodites. a Heatmap of the gene expression pattern of all DMD genes in AFD, ASE, ASK, ASJ, and AWC neurons from previously published single-cell/bulk-sorted neuronal transcriptomic data sets for DMD genes expression that coincide with *ins-39* expression pattern[3–5]. **b** Representative confocal micrographs of a young adult hermaphrodite and male expressing the *ins-39(syb4915)* reporter in wild-type n =15, *dmd-8* mutant (*tm5065*) n =15, *dmd-9* mutant hermaphrodite (*tm4583*) n =12 worms and *dmd-9* mutant males (*tm4583*) n =14 worms. The right panel is the quantification of mean GFP intensity. The pharynx is outlined with a white dashed line. The images were grabbed using different confocal settings in hermaphrodites and males to capture the expression pattern. **c** Quantification of the INS-39 mean GFP intensity using the *ins-39(syb4915)* reporter expression in AFD n =15 wild-type hermaphrodites; n =15 *dmd-9* mutant (*tm4583*) hermaphrodites; n =13 wild-type males; n =14 *dmd-9* mutant (*tm4583*) males, ASK n =14 worms per group in both sexes and ASJ n =14 males per group, AWC n =15 males per group, and ASE n =15 males per group neurons in wild-type males and *dmd-9* mutant (*tm4583*) males. Neurons were identified by co-staining with Vybrant lipophilic dye (DiD) enabling identification of ASK and ASJ, *gcy-8p::GCY-8::wormScarlet* for AFD and *ceh-36p::tagBFP; flp-6p::BFP* for ASE and AWC. **d** Representative confocal micrographs of a L1 hermaphrodite expressing the *ins-39(syb4915)* reporter in AFD neurons in wild-type n =14 and *dmd-9* mutant (*tm4583*) n =15 worms. **e** Quantification of the INS-39 mean GFP intensity from **(d)**. In the box-and-whiskers graph, the center line in the box denotes the median, while the box contains the 25th to 75th percentiles of the dataset, whiskers define the minimum and maximum value with dots showing all points. We performed a two-sided Kruskal-Wallis test for (**b**) and a two-sided Mann-Whitney test (**c**) for each comparison. Scale bars represent 10µm.

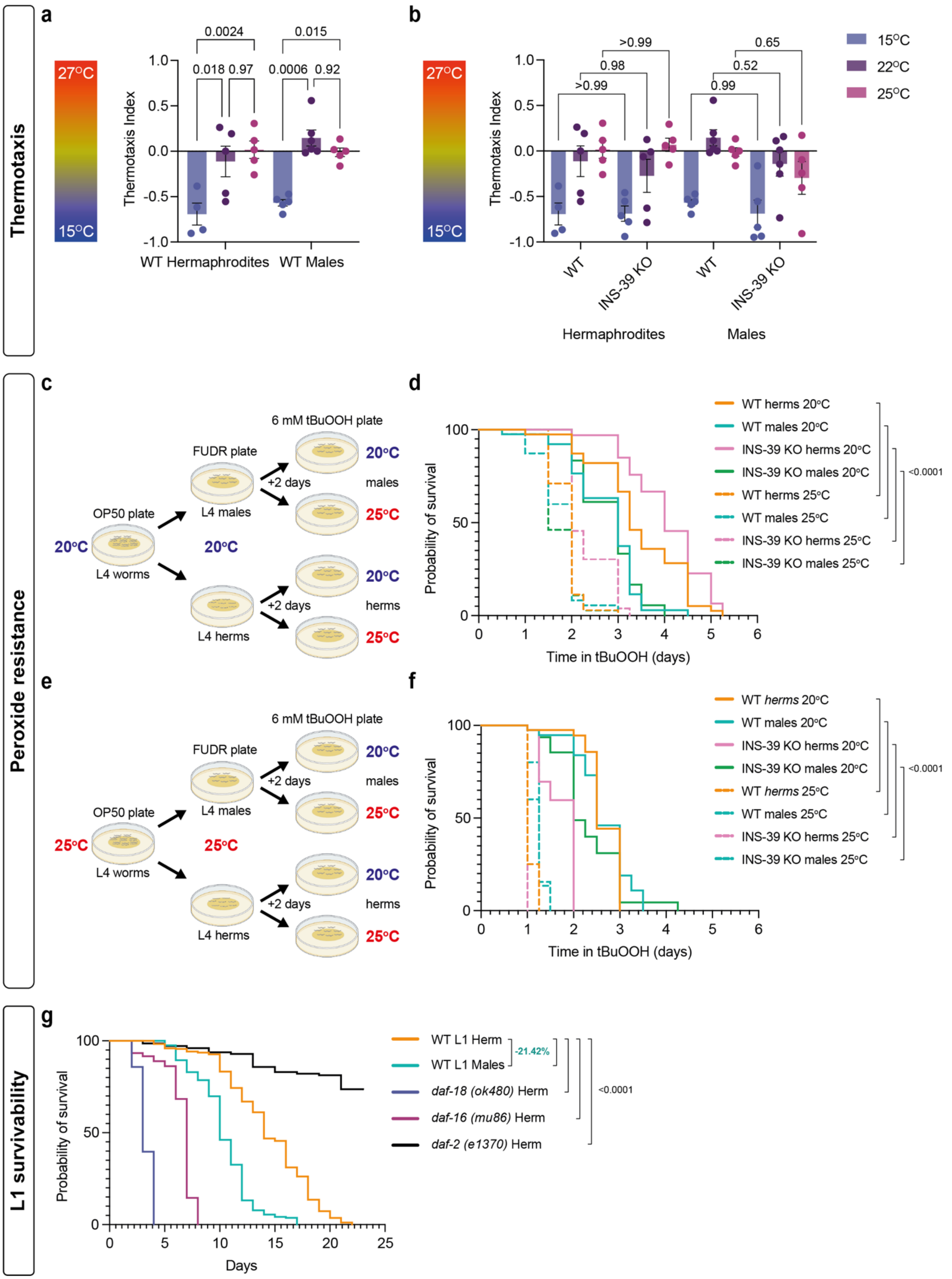

Thermotaxis
a
27°C
15°C
0.0024
0.015
0.018
0.97
0.0006
0.92
Thermotaxis Index
WT Hermaphrodites
WT Males
b
>0.99
0.65
0.98
0.52
>0.99
0.99
15°C
22°C
25°C
WT
INS-39 KO
WT
INS-39 KO
Hermaphrodites
Males
Peroxide resistance
c
6 mM tBuOOH plate
FUDR plate
20°C
+2 days
males
OP50 plate
L4 males
25°C
20°C
20°C
L4 worms
20°C
+2 days
herms
L4 herms
25°C
d
WT herms 20°C
WT males 20°C
INS-39 KO herms 20°C
INS-39 KO males 20°C
WT herms 25°C
WT males 25°C
INS-39 KO herms 25°C
INS-39 KO males 25°C
<0.0001
Probability of survival
Time in tBuOOH (days)
e
6 mM tBuOOH plate
FUDR plate
20°C
+2 days
males
OP50 plate
L4 males
25°C
25°C
25°C
L4 worms
20°C
+2 days
herms
L4 herms
25°C
f
WT herms 20°C
WT males 20°C
INS-39 KO herms 20°C
INS-39 KO males 20°C
WT herms 25°C
WT males 25°C
INS-39 KO herms 25°C
INS-39 KO males 25°C
<0.0001
Probability of survival
Time in tBuOOH (days)
L1 survivability
g
WT L1 Herm
-21.42%
WT L1 Males
daf-18 (ok480) Herm
daf-16 (mu86) Herm
daf-2 (e1370) Herm
<0.0001
Probability of survival
Days

Supplementary Fig. 9. Functional characterization of the role of INS-39. a Quantification of the thermotaxis index in wild-type hermaphrodites and males habituated at 15ºC, 22ºC, and 25ºC for 6 hours. Error bars are standard error of the mean (SEM). We performed a one-way ANOVA test for each comparison. **b** Quantification of the thermotaxis index in wildtype and *ins-39* KO (*ety9)* strain in hermaphrodites and males habituated at 15ºC, 22ºC and 25ºC for 6 hours. Error bars are standard error of the mean (SEM). We performed a one-way ANOVA test for each comparison. For (a, b) n=4 biological repeats for wild-type hermaphrodites habituated at 15ºC, n=5 biological repeats for wild-type hermaphrodites habituated at 22ºC and 25ºC, n= 5 biological repeats for *ins-39* KO (*ety9)* hermaphrodites habituated at 15ºC, 22ºC, and 25ºC. n= 5 biological repeats for Wild-type males and *ins-39* KO (*ety9)* males habituated at 15ºC and 25ºC. n= 6 biological repeats for Wild-type males and *ins-39* KO (*ety9)* males habituated at 22ºC. **c** Schematic of the survival assay on 6mM tBuOOH for (**d**). Created with Biorender.com. **d** Cumulative survival graph on 6mM tBuOOH of wild-type and INS-39 KO hermaphrodites and males grown at 20°C and assayed 20°C or at 25°C. n =40 worms per group. **e** Schematic of the survival assay on 6mM tBuOOH for (**e**). Created with Biorender.com. **f** Cumulative survival graph on 6mM tBuOOH of wild-type and *ins-39* KO (*ety9)* hermaphrodites and males grown at 25°C and assayed 20°C or at 25°C. n =40 worms per group. FUDR, 5-fluoro-2′-deoxyuridine. tBuOOH, tert-butyl hydroperoxide. **g** Cumulative L1 survival graph of wild-type hermaphrodites, wild-type males, *daf-18 (ok480)* hermaphrodites, *daf-16 (mu86)* hermaphrodites, *daf-2 (e1370)* hermaphrodites. Graphs show the composite of three independent experiments. The n for each group in the final datasets was as follows: Wild-type L1 Herm n=2676, Wild-type L1 Male n=2241, *daf-18 (ok480)* Herm n=360, *daf-16 (mu86)* Herm n=515, *daf-2 (e1370)* Herm n=1460. To assess and compare survival rates, the two-sided Kaplan-Meier method was employed. Statistical significance was determined using the Log-rank (Mantel-Cox) test in Prism (GraphPad).

Supplementary References

1. Love, M. I., Huber, W. & Anders, S. Moderated estimation of fold change and dispersion for RNA-seq data with DESeq2. *Genome Biol* **15**, (2014).
2. Kim, B., Suo, B. & Emmons, S. W. Gene Function Prediction Based on Developmental Transcriptomes of the Two Sexes in C. elegans. *Cell Rep* **17**, 917–928 (2016).
3. Godini, R. & Pocock, R. Characterization of the Doublesex/MAB-3 transcription factor DMD-9 in Caenorhabditis elegans. *G3: Genes|Genomes|Genetics* **13**, (2023).
4. Liska, D., Wolfe, Z. & Norris, A. VISTA: Visualizing the Spatial Transcriptome of the C. elegans Nervous System. *bioRxiv* 2023.04.28.538711 (2023) doi:10.1101/2023.04.28.538711.
5. Taylor, S. R. *et al.* Molecular topography of an entire nervous system. *Cell* **184**, 4329-4347.e23 (2021).
