# Supplementary material for: Sex-specific developmental gene expression atlas unveils dimorphic gene networks in C. elegans
Source: Nat Commun. 2024 May 20;15:4273. doi: 10.1038/s41467-024-48369-z (PMC11106331; doi:10.1038/s41467-024-48369-z)
Supplement: Supplementary file 3 — Description of additional supplementary files [file 41467_2024_48369_MOESM3_ESM.pdf]

Description of Additional Supplementary Files

Supplementary Data 1. Male percentages, raw read counts and RINe scores per sample.

Supplementary Data 2. Raw and normalized reads count of male and hermaphrodite genes per sample. Male-enriched and hermaphrodite-enriched genes with their human ortholog and their associated Online Mendelian Inheritance in Man (OMIM) human-disease phenotypes. p-values and adjusted p-values (corrected for multiple testing using the Benjamini and Hochberg method) were calculated by two-sided Wald test for each comparison performed by DESeq2.

Supplementary Data 3. Hermaphrodite and male enriched genes with stage-specific or stage-shared regulation.

Supplementary Data 4: Male-enriched and hermaphrodite-enriched genes comparing differentially expressed genes in both the sexes in this study from L3 to YA with *kim et al, 2016* male and hermaphrodite enriched genes. p-values and adjusted p-values (corrected for multiple testing using the Benjamini and Hochberg method) were calculated by two-sided Wald test for each comparison performed by DESeq2.

Supplementary Data 5. Male-enriched and hermaphrodite-enriched genes with their human ortholog and their associated Online Mendelian Inheritance in Man (OMIM) human-disease phenotypes in L1 and L2 developmental stage. p-values and adjusted p-values (corrected for multiple testing using the Benjamini and Hochberg method) were calculated by two-sided Wald test for each comparison performed by DESeq2.

Supplementary Data 6. Male-enriched and hermaphrodite-enriched transcriptional factor, homeodomain, GPCR, neuropeptide, DM domain, K-channel, ligand-gated ion channels, ionotropic receptor, synaptic vesicle genes and nuclear hormone receptors. p-values and adjusted p-values (corrected for multiple testing using the Benjamini and Hochberg method) were calculated by two-sided Wald test for each comparison performed by DESeq2.

Supplementary Data 7. Transcriptional factors binding site in 1000 bp upstream and 500 bp downstream of the transcription start site of *ins-39.* p-values were computed from the matrix, according to RSAT matrix-based programs sing either a Bernoulli or a Markov-chain model.

Supplementary Data 8. List of strains, reagent and resource used in this study.
