# Supplementary material for: Sex-specific developmental gene expression atlas unveils dimorphic gene networks in C. elegans
Source: Nat Commun. 2024 May 20;15:4273. doi: 10.1038/s41467-024-48369-z (PMC11106331; doi:10.1038/s41467-024-48369-z)
Supplement: Supplementary file 12 — Reporting Summary [file 41467_2024_48369_MOESM12_ESM.pdf]

nature portfolio

Corresponding author(s): Oren-Suissa

Last updated by author(s): Mar 17, 2024

Reporting Summary

Nature Portfolio wishes to improve the reproducibility of the work that we publish. This form provides structure for consistency and transparency in reporting. For further information on Nature Portfolio policies, see our Editorial Policies and the Editorial Policy Checklist.

Statistics

For all statistical analyses, confirm that the following items are present in the figure legend, table legend, main text, or Methods section.

n/a	Confirmed	
☐	☒	The exact sample size (*n*) for each experimental group/condition, given as a discrete number and unit of measurement
☐	☒	A statement on whether measurements were taken from distinct samples or whether the same sample was measured repeatedly
☐	☒	The statistical test(s) used AND whether they are one- or two-sided<br>*Only common tests should be described solely by name; describe more complex techniques in the Methods section.*
☒	☐	A description of all covariates tested
☐	☒	A description of any assumptions or corrections, such as tests of normality and adjustment for multiple comparisons
☐	☒	A full description of the statistical parameters including central tendency (e.g. means) or other basic estimates (e.g. regression coefficient) AND variation (e.g. standard deviation) or associated estimates of uncertainty (e.g. confidence intervals)
☐	☒	For null hypothesis testing, the test statistic (e.g. *F*, *t*, *r*) with confidence intervals, effect sizes, degrees of freedom and *P* value noted<br>*Give P values as exact values whenever suitable.*
☒	☐	For Bayesian analysis, information on the choice of priors and Markov chain Monte Carlo settings
☒	☐	For hierarchical and complex designs, identification of the appropriate level for tests and full reporting of outcomes
☒	☐	Estimates of effect sizes (e.g. Cohen's *d*, Pearson's *r*), indicating how they were calculated

Our web collection on statistics for biologists contains articles on many of the points above.

Software and code

Policy information about availability of computer code

Data collection	WormLab®, NextSeq500, Zeiss LSM 880 confocal microscope , Zeiss stemi 508 mounted with Zeiss Axiocam ERc 5s , NanoDrop™ One/OneC Microvolume UV-Vis Spectrophotometer, StepOnePlus™ Real-Time PCR System, Qubit Fluorometer Model #4, Bioanalyzer, BIOSORTER® flow cytometer, 2200 TapeStation Nucleic Acid system
Data analysis	For MARSseq analysis UTAP pipeline was used (https://utap.wexac.weizmann.ac.il/), ImageJ/Fiji software, version 2.3.0/1.53q, Zen (Zeiss, v2.3), Wormlab (MBF Biosciences), Prism 9 (GraphPad) version (9.5.0), Illustrator V 25.0.1, Flow pilot III BioSorter profiler V 3.0.8.3 , FlowJo V 10.8.1.

For manuscripts utilizing custom algorithms or software that are central to the research but not yet described in published literature, software must be made available to editors and reviewers. We strongly encourage code deposition in a community repository (e.g. GitHub). See the Nature Portfolio guidelines for submitting code & software for further information.

Data

Policy information about availability of data

All manuscripts must include a data availability statement. This statement should provide the following information, where applicable:

- Accession codes, unique identifiers, or web links for publicly available datasets
- A description of any restrictions on data availability
- For clinical datasets or third party data, please ensure that the statement adheres to our policy

The authors declare that all data generated or analyzed during this study are included in this published article (and its supplementary information files). The sequence data generated in this study have been deposited in NCBI database and are available under BioProject GSE237937 (https://www.ncbi.nlm.nih.gov/geo/query/acc.cgi?acc=GSE237937). All raw data generated or analyzed in this study are included in the manuscript along with supplementary information files and Source Data file. Source data are provided with this paper. The RNA-seq datasets associated with this article are included in Supplementary Data 2. RNA-seq reads were and mapped to the C. elegans reference genome WBcel235 (https://www.ncbi.nlm.nih.gov/datasets/genome/GCF_000002985.6/)

Research involving human participants, their data, or biological material

Policy information about studies with human participants or human data. See also policy information about sex, gender (identity/presentation), and sexual orientation and race, ethnicity and racism.

Reporting on sex and gender	N/A
Reporting on race, ethnicity, or other socially relevant groupings	N/A
Population characteristics	N/A
Recruitment	N/A
Ethics oversight	N/A

Note that full information on the approval of the study protocol must also be provided in the manuscript.

Field-specific reporting

Please select the one below that is the best fit for your research. If you are not sure, read the appropriate sections before making your selection.

☒ Life sciences ☐ Behavioural & social sciences ☐ Ecological, evolutionary & environmental sciences

For a reference copy of the document with all sections, see nature.com/documents/nr-reporting-summary-flat.pdf

Life sciences study design

All studies must disclose on these points even when the disclosure is negative.

Sample size	No sample size calculations were performed. The sample sizes in the study were chosen based on prior experience, reasonable replication, and standards in the field. Wherever possible, animal numbers were kept at 12-20 per sex/genotype. Sample size was not less than 10 animals per group. For our assays, the sample size that is desired for observing a phenotype is 12-15 animals per group. When genetic backgrounds are challenging for growth and maintenance, we used a sample size lower than 15 but not lower than 10.
Data exclusions	No data were excluded
Replication	Wild-type and untreated controls were done separatly for each experiment and as such independently confirm the same findings and can be consisered as replicates. For all experiments, mutant positive-phenotypes were repeated at least 3 times independently.
Randomization	Animals for each experiment were picked randomly from large population plates.
Blinding	The experimenter was not-blinded to the genotype or sex of the animals tested.

Reporting for specific materials, systems and methods

We require information from authors about some types of materials, experimental systems and methods used in many studies. Here, indicate whether each material, system or method listed is relevant to your study. If you are not sure if a list item applies to your research, read the appropriate section before selecting a response.

Materials & experimental systems

n/a	Involved in the study
☒	☐ Antibodies
☒	☐ Eukaryotic cell lines
☒	☐ Palaeontology and archaeology
☐	☒ Animals and other organisms
☒	☐ Clinical data
☒	☐ Dual use research of concern
☒	☐ Plants

Methods

n/a	Involved in the study
☒	☐ ChIP-seq
☐	☒ Flow cytometry
☒	☐ MRI-based neuroimaging

Animals and other research organisms

Policy information about studies involving animals; ARRIVE guidelines recommended for reporting animal research, and Sex and Gender in Research

Laboratory animals	C. elegans, N2 bristol strain, hermaphrodites and males of various developmental stages. All the strains used in this study are listed in the supplementary material file.
Wild animals	none
Reporting on sex	Sexual identity was considered and fully reported.
Field-collected samples	No field collected samples were used in the study
Ethics oversight	Ethical approval is not required for work with soil nematodes

Note that full information on the approval of the study protocol must also be provided in the manuscript.

Plants

Seed stocks	N/A
Novel plant genotypes	N/A
Authentication	N/A

Flow Cytometry

Plots

Confirm that:

☒ The axis labels state the marker and fluorochrome used (e.g. CD4-FITC).

☒ The axis scales are clearly visible. Include numbers along axes only for bottom left plot of group (a 'group' is an analysis of identical markers).

☒ All plots are contour plots with outliers or pseudocolor plots.

☒ A numerical value for number of cells or percentage (with statistics) is provided.

Methodology

Sample preparation	Sample used were whole C. elegans organism. C. elegans strains ins-39p::gfp or him-5 (control) were grown and maintained at 20°C. L1 synchronized nematode cultures were obtained by standard bleaching protocols.
Instrument	Worms sorting was performed on a BioSorter equipped with a 250 FOCA and S-basal as the sheath fluid.
Software	BIOSORTER FlowPilot™ software

Cell population abundance	NO cells were involved. L1-arrested C. elegans larvae were collected in 50 ml conical tubes and were resuspended in S-basal at a final density of about 2000 worms/ml.
Gating strategy	Worms were gated based on time of flight in the flow cytometer flow cell and extinction at 488 nm and then sub-gated based on time of flight vs. GFP intensity peak height at 512 nm. This has been clearly stated in the figure legend.

☒ Tick this box to confirm that a figure exemplifying the gating strategy is provided in the Supplementary Information.
